# Inter-organ correlations in inflammation regulation: a novel biological paradigm in a murine model

**DOI:** 10.25122/jml-2024-0246

**Published:** 2025-01

**Authors:** Yehudit Shabat, Devorah Rotnemer-Golinkin, Lidya Zolotarov, Yaron Ilan

**Affiliations:** 1 Faculty of Medicine, Hebrew University, Jerusalem, Isreal; 2 Department of Medicine, Hadassah Medical Center, Jerusalem, Israel

**Keywords:** inflammation, information transfer, Concanavalin A, immune hepatitis, Cona, Concanavalin A, AST, Aspartate Aminotransferase, ALT, Alanine Aminotransferase, WQT, Weak Quantum Theory

## Abstract

Interactions between immune system constituents are mediated through direct contact or the transfer of mediators. The study aimed to assess the correlation between system components and out-of-body signals in a model of liver inflammation. In the first experiment, mice injected with Concanavalin A (ConA) were housed in a cage with a tube on top containing healthy livers or livers harvested from mice injected with ConA. In the second experiment, mice were housed in a cage with a tube that contained splenocytes harvested from naïve donors or from naïve donors treated in vitro with dexamethasone. Mice were tested for serum aspartate aminotransferase (AST) and alanine aminotransferase (ALT) levels. External whole livers and spleens influenced the immune-mediated inflammatory response of mice. When ConA-injected mice were housed in cages with tubes containing livers harvested from naïve mice, ALT serum levels were significantly reduced. ALT serum levels were significantly elevated when mice were kept in cages with a tube containing livers harvested from ConA-injected mice. In the second part of the experiment, mice injected with ConA and housed in cages with a tube on top that contained splenocytes harvested from naïve donors had increased ALT levels. Similarly, mice with tubes containing splenocytes from dexamethasone-treated naïve donors also showed elevated ALT levels. The data suggest that correlations between immune system constituents can be established using out-of-body whole livers or spleens without contact or transfer of mediators.

## INTRODUCTION

Complex biological systems require information transfer at several levels, ranging from subcellular and cellular to the whole organs [1-3]. In classical immunology, interactions between immune system components occur through direct communication between cells or immune mediators (e.g., cytokines, chemokines) secreted by different cellular constituents [4-6]. While these mechanisms are well established, they fail to explain all the effects observed under various conditions where the immune system participates. The current 'direct touch' paradigm cannot fully explain the different interactions between immune cells. Similarly, several genotype-phenotype-epigenetic immune interfaces require more than one pathway to be thoroughly described. Correlation between immune system components was recently described in an isolated system where out-of-body lymphocytes were impacted by external triggers [7,8]. However, a correlation between whole organs to alleviate or promote inflammatory processes has yet to be established.

The present study aimed to describe a schematic model for an alternative pathway in the immune system in which correlation between organs alters the inflammatory response. According to this model, this does not occur through direct contact or the transfer of mediators.

## MATERIAL AND METHODS

### Animals

Male C57BL/6 mice (11–12 weeks old) were obtained from Harlan Laboratories (Jerusalem, Israel) and maintained in the Animal Core of the Hadassah-Hebrew University Medical School. Mice were administered standard laboratory chow and water *ad libitum* and kept in a 12-hour light/dark cycle. All animal experiments were conducted following the guidelines of the Hebrew University Faculty of Medicine Hadassah Institutional Committee for Care and Use of Laboratory Animals. The study complies with all regulations for animal welfare. Animals in all studies were anesthetized using ketamine (80-100 mg/kg intraperitoneally [IP]) and xylazine (5-10 mg/kg IP).

### Study design and experimental groups

This study investigated the correlation between organs in an isolated system and their impact on liver inflammation. Two sets of experiments were conducted, with four groups of mice being studied in the first experiment (Table 1).

**Table 1:** Experimental groups (first experiment)

Group *n* = 4 (A, B) n = 6 (C, D)	ConA	Tube on cage
A	+	-
B	-	-
C	+	6 healthy livers in a tube
D	+	6 livers from mice injected with ConA

In Group A (*n* = 4), mice were injected intraperitoneally with Concanavalin A (ConA, 500 mg/mouse) to induce immune-mediated liver injury, as described in previous studies [9,10]. Mice in Group B (*n* = 4) were naïve healthy mice. In Group C (*n* = 6), mice were injected with ConA and housed in a cage with a tube on top containing six healthy livers. Mice in Group D (*n* = 6) were injected with ConA and housed in a cage with a tube containing six livers harvested from mice injected with ConA.

The second experiment included six groups of mice as a follow-up to the first study (Table 2, *n* = 5/group). All mice were injected with ConA. Mice in Group A were not treated further and were housed in a control cage. Mice in Group B were orally treated with dexamethasone (0.35 mg/ mouse) two hours before ConA injection. Mice in Group C were housed in a cage with a tube that contained splenocytes harvested from five naïve donors. Mice in Group D were kept in a cage with a tube containing splenocytes harvested from five naïve donors treated in vitro with dexamethasone (1 mg/spleen). Groups E and F were identical to Groups C and D in experiment one. Mice in Group E were housed in a cage with a tube containing five livers harvested from five naïve donors. Mice in Group F were housed in a cage with a tube on top containing five livers harvested from five donors injected with ConA.

**Table 2:** Experimental groups (second experiment)

Group *n* = 5	ConA	Dexamethasone	Tube on cage
A	+	-	-
B	+	+	-
C	+	-	Splenocytes harvested from five naïve donor
D	+	-	Splenocytes harvested from five naive donors treated in vitro with dexamethasone
E	+	-	Livers harvested from five naïve donors
F	+	-	Livers harvested from five mice injected with ConA

All mice from all groups were tested for serum aspartate aminotransferase (AST) and alanine aminotransferase (ALT) levels 16 hours following ConA injection, using an automatic analyzer for both experiments. At the end of the experiment, mice were anesthetized with ketamine and xylazine, and serum was collected.

### Statistical analysis

The Student's *t*-test was used for data analysis. Individual data from all mice across both experiments are provided in the supplementary file (Table S1).

Supplementary File

## RESULTS

A schematic experiment was designed in which mice were injected with ConA and housed in cages with sealed tubes placed on top. These tubes contained livers or spleens harvested from naïve donors, ConA-injected donors, or dexamethasone-treated donors. This system enabled the study of out-of-body correlations between immune system constituents by targeting specific organs to assess their ability to regulate immune-mediated liver inflammation in an isolated system.

### Effect of healthy and injured liver tissues on immune-mediated liver inflammation

**Figure 1:**
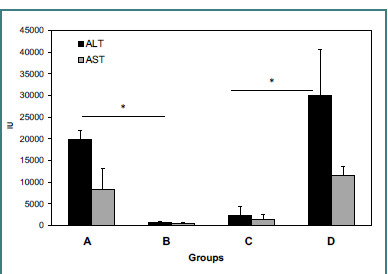
Effect of external liver tissues on ConA-induced liver inflammation. Serum ALT and AST levels (mean ± SD) were measured in four groups of mice. High ALT serum levels were measured in Group A, injected with ConA, and compared with naïve controls in Group B (P < 0.005). When ConA-injected mice were housed in cages with tubes containing livers harvested from naïve mice (Group C), a statistically significant reduction in ALT serum levels was noted (P < 0.005, for C vs. A). When mice were housed in cages with a tube containing livers harvested from ConA-injected mice (Group D), a statistically significant increase in ALT serum levels was noted (P < 0.005, for D vs. B, P = NS for D vs. B). A similar effect was noted for AST serum levels.

A correlation was observed in the isolated system, demonstrating that exposure to external liver tissues influenced the ConA-induced inflammatory response in the liver. Figure 1 shows ALT and AST serum levels in all groups of mice as markers of inflammatory response in the liver. Mice injected with ConA (Group A) had significantly elevated ALT levels compared to naïve controls (Group B) (19,868 ± 1,982 IU vs. 633 ± 354 IU, *P* < 0.005). When ConA-injected mice were housed in cages with tubes containing livers harvested from naïve mice (Group C), a statistically significant reduction in ALT serum levels was noted (2,377+1,976 IU, *P* < 0.005, vs. Group A). In contrast, in mice housed with tubes containing livers harvested from ConA-injected donors (Group D), a statistically significant increase in ALT serum levels was observed (29,922 ± 10,652 IU, *P* <0.005, vs. Group B; *P* = NS for D vs. B). A similar effect was noted for AST serum levels.

### Correlation between immune system constituents induced by naïve and steroid-treated splenocytes or healthy and injured livers

**Figure 2:**
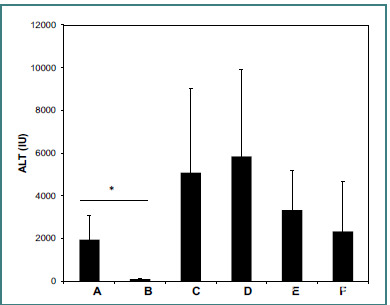
Effect of external tissues on inflammation. A schematic model of an isolated system was used to evaluate the ConA-mediated inflammatory response in the liver. Six groups of mice (n = 5/group) were studied. All mice were injected with ConA, and serum ALT levels were measured 16 hours post-injection (mean ± SD). Mice in control Group A were injected with ConA and exhibited high levels of ALT. In contrast, Group B, treated with dexamethasone, showed significant improvement in liver enzyme levels (P < 0.005). Mice in Group C were injected with ConA and housed in a cage with a tube on top that contained splenocytes harvested from five naïve donors. ALT levels were increased (P = NS, for C vs. A). An increase in serum ALT levels was demonstrated in Group D, injected with ConA and housed in a cage with a tube containing splenocytes harvested from five naïve donors treated with dexamethasone (P = NS, for D vs. A). Mice in Groups E and F were injected with ConA and housed in a cage with a tube containing five livers harvested from five naïve donors (Group E) or those injected with ConA (Group F). Experiment one showed these conditions identical to mice in groups C and D. ALT levels increased compared to that in Group A in both groups (P = NS for E vs. A and F vs. A).

A schematic model was established to assess ConA-mediated inflammatory response in the liver in an isolated system. Figure 2 shows serum levels of ALT across all groups as an indicator of liver inflammation. Mice in control Group A, injected with ConA, had high levels of ALT. In contrast, mice in Group B, treated with dexamethasone, had significant improvements in liver enzyme levels (1,791 ± 764 IU vs. 338 ± 119 IU for Groups A and B, respectively, *P* < 0.005). Mice in Group C, injected with ConA and housed in a cage with a tube containing splenocytes from five naïve donors, had an increase in ALT levels (4,118 ± 2,785 IU, *P* = NS vs. Group A). Similarly, mice in Group D, injected with ConA and housed with a tube containing splenocytes from five naïve donors treated with dexamethasone, showed an increase in ALT levels (5,760 ± 5,789 IU, *P* = NS vs. Group A). Mice in Groups E and F were injected with ConA and housed in a cage with a tube containing five livers harvested from either naïve donors (Group E) or those injected with ConA (Group F). Experiment one showed these conditions identical to mice in Groups C and D. ALT levels increased compared to that in Group A in both groups (2894 ± 1432 IU and 2029 ± 1514 IU for Groups E and F, respectively, *P* =NS for E vs. A and F vs. A).

The lack of statistical significance for the differences between mice in Groups C to F and Group A was due to a sizeable intra-group variability, suggesting a potential recurring pattern within the experimental system. However, treating each mouse as an independent experimental unit allowed for an alternative analysis. In Group C, four mice had a significant increase in ALT levels (>2300 IU). Similarly, in Group D, a significant increase in ALT levels was observed in four mice, and an increase was observed in three mice in Group E.

A statistical comparison of these subgroups with Group A showed a highly significant difference (*P* = 0.05 for mice marked C2, C3, C4, C5; *P* = 0.04 for mice marked D1, D2, D3, D5; and *P* = 0.04, for mice marked E2, E3, E4, vs. Group A). These data suggest that correlations between immune system constituents and external organs can occur in an isolated system, affecting inflammatory responses without direct contact or known mediator transfer.

## DISCUSSION

In this study, we established an isolated system in which correlations between immune system constituents and out-of-body livers or spleens were observed. Information transfer in the immune system results from contact between immune system constituents or the transfer of mediators secreted by immune cells [11,12]. In the model described here, an association between components of the immune system and the out-of-body organ occurred across a distance of 10-20 cm, resulting in ConA-induced regulation of immune-mediated liver inflammation.

The data shown here illustrate an alternative pathway for immune-mediated regulation of the liver's inflammatory state in vivo, without direct contact or transfer of mediators. In the experiment described here, a measurable effect on the inflammatory condition of the liver was detected in mice injected with ConA, which were exposed to healthy or sick livers or spleens placed on top of their cages.

The absence of consistency in results obtained from individual mice within the same group and changes in correlation in opposite directions between individual animals and the two experiments were observed. Partial consistency may result from varying factors that affect immune pathways and play different roles in individual mice. The data are presented as the mean for all mice in each group. Results for each mouse suggest an inherent repeating pattern for some mice in each group.

Correlation between organs exists at several levels. Some of these correlations may be affected inversely in different individuals. The statistical significance of the results could have been higher due to the low number of mice per group being studied. High intra-group variabilities further underscored the need for statistical significance of the results. It was proposed that an average value is unsuitable for application to biological systems [13,14].

Fractal physiology concepts can be used to describe alternative immune pathways in which variability is more physiologically relevant than an average value. In physics, objects are considered generic, wherein different objects are assumed to be identical and progress in distinct state spaces [15]. In contrast, like other biological systems, the immune system can exhibit randomness, historicity, and contextuality under specific conditions [14,16-25]. The concepts of order and randomness have been proposed to be inherent to the structural and dynamical rules of biological systems [8,14,17-31]. It was suggested that variation is a fundamental characteristic in biology and can be used to correct system malfunctions [25-27,32-72]. It is based on the notion that in any biological system, each organism is different, and each individual may undergo unpredictable and random changes [14,16]. Bio-entanglement has been proposed to translate several models of information transfer into biological phenomena [73]. Randomness and variability in such systems can be conveyed using expressions of symmetry change. The information generated in the system characterized here may be inherently random to some extent [26,27,54,58,73]. Generating identical random states for all levels of biological systems tested is challenging and may have beneficial effects [34-51,64-70]. This phenomenon might explain part of the system's incoherence and may account for its lack of complete adherence and repeatability.

The data from the present study does not describe an apparent natural phenomenon and may result from the probability of several events. As only actual states are observed following measurement, the quantifiable effect on the immune system displays a sum of probabilities resulting from the new state generated in this system in each animal. Exposure to stress is known to directly impact the immune system's characteristics [59]. It is not a pure statistical route but a probability induced by a measurable system or parameters. Using an isolated system allowed our protocol to succeed without post-selection with reasonable consistency. Understanding the different levels of randomness that may be inherent to a system, partly resulting from multiple environmental factors [74], is essential for future studies and the application of this model.

The ambiguity of physical properties is inherent in quantum physics [8,75]. In physics, a process affects the system being studied [54] at the atomic level in isolated systems. Actual measurements in a system may not reflect reality but rather create a new certainty based on selection, according to probability [76-78]. Broadening the spectrum of theoretical concepts beyond a local-causality model toward a non-local one has been proposed [79]. Such a model can be derived from a generalized and somewhat weaker version of the weak quantum theory (WQT). WQT predicts entanglement between system elements if two variables or observables are complementary, the first describing a global and the second describing the system's local aspects [79]. A similar phenomenon may underline observations in biological systems in general and particularly in the immune system [73,80-82].

Physiological systems are generally information-preserving [76]. The present study did not involve brain-computer interfaces (BCIs) or brain-brain interfaces (BBIs), suggesting that these are not mandatory for organ correlations. A non-invasive BCI-mediated information transfer has been described in which brain functions are translated to generate computer commands. Studies have demonstrated brain-to-brain communication (BBI), where a human volunteer’s brain activity successfully stimulated a rat’s motor cortex, triggering tail movements [83]. These findings support the feasibility of computer-mediated neural interfaces.

The limitations of this study involve the small number of groups and the need to determine the biological effect on inflammatory immune cells and molecules to dissect the underlying mechanism further.

## CONCLUSION

In summary, the present data describe an isolated system in which correlations can occur between immune system constituents and out-of-body organs without direct contact or transfer of mediators. These results support the existence of alternative pathways for correlations between different constituents of the immune system and the target organs. These correlations may underlie some of the observables in which the immune system is involved but cannot be explained using the classical immune pathways based on direct contact or transfer of mediators. These findings shed light on additional ways to transfer information between immune system constituents. The data also suggest alternative ways of explaining immune memory. Further dissection of these alternative immune pathways may enable their use for diagnostic and therapeutic purposes to be performed on out-of-body organs to treat immune-associated disorders.

## Data Availability

Data from all experiments is included in the supplementary file.
