# Supplementary material for: Inter-organ correlations in inflammation regulation: a novel biological paradigm in a murine model
Source: J Med Life. 2025 Jan;18(1):67–72. doi: 10.25122/jml-2024-0246 (PMC11891616; doi:10.25122/jml-2024-0246)
Supplement: Supplementary file 1 [file JMedLife-18-067-s001.pdf]

Supplementary Material

Table S1. Experiment 1

	Mice	ALT	Average/ SD	AST	Average/ SD
**A**	A1	20910	19868	12810	8423
	A2	16950		3030	
	A3	20340	1982	5610	4858
	A4	21270		12240	
**B**	B1	339	633	318	459
	B2	342		390	
	B3	795	354	462	150
	B4	1056		666	
**C**	C1	4920	2377	2769	1471
	C2	438		540	
	C3	2472	1976	1278	1011
	C4	3630		2247	
	C5	423		522	
**D**	D1	32100	29922	12510	11628
	D2	23580		13470	
	D3	47700	10652	13110	1971
	D4	22860		9930	
	D5	23370		9120	

Table S2. Experiment 2

	Mice	Dilution	Value	ALT IU	Average	SD		Mice	Dilution	Value	AST IU	Average	SD
A	**A1**	10	325	3250			A	**A1**	10	237	2370		
	**A2**	10	177	1770				**A2**	10	211	2110		
	**A3**	10	63.1	631				**A3**	10	78.7	787		
	**A4**	10	294	2940				**A4**	10	251	2510		
	**A5**	10	110	1100	1938.20	1136.23		**A5**	10	118	1180	1791.40	764.08
B	**B1**	5	17.6	88			B	**B1**	5	63.5	317.5		
	**B2**	5	17.9	89.5				**B2**	5	101	505		
	**B3**	5	12.6	63				**B3**	5	47.9	239.5		
	**B4**	5	15	75				**B4**	5	44.1	220.5		
	**B5**	5	23	115	86.10	19.41		**B5**	5	82.1	410.5	338.60	119.45

© 2025 by the authors. This article is an open access article distributed under the terms and conditions of the Creative Commons Attribution (CC BY 4.0) license.

Table S2. Continued. Experiment 2

	Mice	Dilution	Value	ALT IU	Average	SD		Mice	Dilution	Value	AST IU	Average	SD
C	**C1**	10	110	1100			C	**C1**	10	105	1050		
	**C2**	10	908	9080				**C2**	20	343	6860		
	**C3**	10	951	9510				**C3**	20	364	7280		
	**C4**	10	320	3200				**C4**	10	291	2910		
	**C5**	10	251	2510	5080.00	3924.43		**C5**	10	249	2490	4118.00	2785.64
D	**D1**	10	273	2730			D	**D1**	10	207	2070		
	**D2**	20	562	11240				**D2**	40	175	7000		
	**D3**	10	371	3710				**D3**	10	244	2440		
	**D4**	10	238	2380				**D4**	10	189	1890		
	**D5**	10	919	9190	5850.00	4079.30		**D5**	40	385	15400	5760.00	5789.70
E	**E1**	10	175	1750			E	**E1**	10	169	1690		
	**E2**	10	274	2740				**E2**	10	267	2670		
	**E3**	10	480	4800				**E3**	20	250	5000		
	**E4**	10	572	5720				**E4**	20	177	3540		
	**E5**	10	159	1590	3320.00	1853.69		**E5**	10	157	1570	2894.00	1423.49
F	**F1**	10	102	1020			F	**F1**	10	121	1210		
	**F2**	10	88.2	882				**F2**	10	93.5	935		
	**F3**	10	648	6480				**F3**	20	234	4680		
	**F4**	10	189	1890				**F4**	10	171	1710		
	**F5**	10	136	1360	2326.40	2354.24		**F5**	10	161	1610	2029.00	1514.32

© 2025 by the authors. This article is an open access article distributed under the terms and conditions of the Creative Commons Attribution (CC BY 4.0) license.
